# Effect of cross exercise on quadriceps acceleration reaction time and subjective scores (Lysholm questionnaire) following anterior cruciate ligament reconstruction

**DOI:** 10.1186/1749-799X-4-2

**Published:** 2009-01-30

**Authors:** Maria G Papandreou, Evdokia V Billis, Emmanouel M Antonogiannakis, Nikos A Papaioannou

**Affiliations:** 1 School of Physiotherapy, Faculty of Health Sciences, Technological Education Institute (T.E.I) of Athens, Athens, Greece; 2 School of Physiotherapy, Faculty of Health Sciences, Aigio, Technological Education Institute (T.E.I) of Patras, Patras, Greece; 3 2nd Orthopaedic Department of 401 General Military Hospital, Athens, Greece; 4 Research Laboratory of Musculoskeletal System, University of Athens, Athens, Greece

## Abstract

**Background:**

Anterior cruciate ligament (ACL) injury or reconstruction can cause knee impairments and disability. Knee impairments are related to quadriceps performance – accelerated reaction time (ART) – and disability to performance of daily living activities which is assessed by questionnaires such as the Lysholm knee score. The purposes of this study were to investigate the effect of cross exercise, as supplementary rehabilitation to the early phase of ACL reconstruction: a) on quadriceps ART at the angles 45°, 60° and 90° of knee flexion and, b) on the subjective scores of disability in ACL reconstructed patients.

**Methods:**

42 patients who underwent ACL reconstruction were randomly divided into 3 groups, two experimental and one control. All groups followed the same rehabilitation program. The experimental groups followed 8 weeks of cross eccentric exercise (CEE) on the uninjured knee; 3 d/w, and 5 d/w respectively.

Quadriceps ART was measured at 45°, 60° and 90° of knee flexion pre and nine weeks post-operatively using an isokinetic dynamometer. Patients also completed pre and post operatively the Lysholm questionnaire whereby subjective scores were recorded.

**Results:**

Two factor ANOVA showed significant differences in ART at 90° among the groups (F = 4.29, p = 0.02, p < 0.05). Post hoc Tukey HSD analysis determined that the significant results arose from the first experimental group in comparison to the control (D = -0.83, p = 0.01). No significant differences were revealed at 45° and 60°.

Significant differences were also found in the Lysholm score among the groups (F = 4.75, p = 0.01, p < 0.05). Post hoc analysis determined that the above significant results arose from the first experimental group in comparison with the control (D = 7.5, p < 0.01) and from the second experimental in comparison with the control (D = 3.78, p = 0.03).

**Conclusion:**

CEE showed improvements on quadriceps ART at 90° at a sequence of 3 d/w and in the Lysholm score at a sequence of 3 d/w and 5 d/w respectively on ACL reconstructed patients.

## Background

It is well established that patients who have an ACL rupture demonstrate physical impairments and disability related to the injury [1-3]. Despite conservative treatment most patients will undergo ACL reconstruction. Traditionally, clinicians have utilized various outcomes as criteria to assess impairment and disability following ACL injury [2,3].

Impairments following ACL injury are functional (anterior displacement of the tibial relative to the femur) and physiologic (range of motion, muscle performance and pain). These can be measured by the KT-1000 knee arthrometer, goniometer, isokinetic muscle tests, and visual analogue scale of pain [3].

Disability following ACL injury is related to performance of daily activities, leisure time activities, or sports activities and has traditionally been measured with valid questionnaires, such as the Lysholm and Cincinnati knee scores, and functional knee tests [3,4].

Quadriceps muscle dysfunction- weakness or reduced accelerated reaction time- is recognized as significant complication following ACL reconstruction [5-8]. Quadriceps muscle activity causes an anterior translation of the tibia approximately in the range from 20° to 60° or 75° of knee flexion with maximal tibia displacement occurring at 45° (quadriceps mechanical disadvantage) and less at 90° of flexion (maximum strength produced) [9-14].

Despite, the plethora of the progressive and accelerated exercise programs for ACL reconstructed patients, long term impairments and quadriceps deficiency often persists [5,15,16].

Therefore, identifying an exercise protocol such as cross exercise (CE), as an adjunct to traditional ACL rehabilitation program may facilitate rehabilitation strategies and thereby maximize functional outcomes.

CE is referred to the contralateral limb, by increasing strength in the homologous muscle of the untrained limb, without directly involving the latter in the motor activity [17]. Several neural mechanisms have been proposed for CE including diffusion of impulses between hemispheres, coactivation via bilateral corticospinal pathways, postural stabilization and the cerebral cortex theory which has been referred to as the most dominant mechanism [17,18]. It is explained by the theory that during the voluntary contraction of a muscle on the trained side is produced a facilitation effect on the same motor point in the opposite side of the cerebral cortex [17,18]. This is also explained anatomically by the fact that 10% of the corticospinal fibers enter in the lateral and anterior corticospinal tract of the trained side, whereas the remaining fibers cross to the opposite side of it through diffusion of impulses [17-19].

Benefits of CE have been well established on quadriceps strength improvement in healthy subjects [19-23]. As far as the type of CE is concerned, eccentric exercise has been found to be superior to isometric and concentric exercise [19,24,25]; and has had the greatest effect on quadriceps strength improvement accounting for the greater increases in eccentric and isometric forces [19,24-26]. However, an intraspinal mechanism is probably more likely to mediate CE especially in studies that observed remarkably greater CE using eccentric contractions [17,20,22,26].

In addition, it would be suggested that eccentric contractions are associated with unique motor unique activation strategies by the nervous system and that the process of inducing CE may be different for training with concentric and isometric contractions [19,24-27].

Thereby, eccentric exercise benefits have been well established in the literature as the muscle forces which are produced during muscle lengthening are extremely high, despite the requisite low energetic cost [27].

As far as the frequency of CE is concerned, there is no consensus across the literature which supports an association between the training frequency and the benefits of CE [17]. However, most studies have used as the most appropriate frequency three days per week [17,19,20,22,28].

Limited studies have reported the effect of cross exercise in patients following knee reconstruction [28,29]. Papandreou et al [28] have shown that cross eccentric exercise has been proved to be a useful mechanism in strengthening the quadriceps muscle on the ACL reconstructed knee by training the uninjured knee, at knee angles at 45° and 90° of knee motion at a sequence of 3 d/w, in the early rehabilitation phase of ACL reconstruction.

Based on the above, it is not unreasonable to assume that the use of cross eccentric exercise more than three days per week-such as five days per week- as an adjunct to traditional ACL rehabilitation program might have an enhanced effect on CE, assist rehabilitation methods and thereby maximize quadriceps functional outcomes, in the early ACL postoperative period.

Thus, the primary purpose of this study was to investigate the effect of cross eccentric exercise (CEE), applied three and five days per week, on the quadriceps accelerated reaction time at the angles 45°, 60° and 90° of knee flexion, in the early rehabilitation phase of ACL reconstructed knee.

The secondary purpose was to investigate the effect of CEE, applied three and five days per week, on the subjective scores of disability questionnaire, in the early rehabilitation of ACL reconstructed patients.

## Materials and methods

Subjects were randomly divided into 3 groups (2 experimental and 1 control). Measurements were taken three days before the operation and nine weeks following the ACL reconstruction procedure.

### Subjects

Forty two patients, all male volunteer-soldiers from the Greek army participated in this study. All patients had sustained a unilateral ACL rupture and were randomly assigned (by coin flip) into three groups, two experimental and one control, comprising fourteen subjects each.

ACL rupture was confirmed by the same orthopaedic surgeon, as well as by MRI examination

In order to assure group homogeneity, all subjects required the following inclusion criteria: a) their ages ranged between 20–25 years, b) complete rupture of ACL without combined injuries that needed reconstruction, c) the side-to-side difference of tibial anterior translation (SD) was greater than 3 mm on the KT1000 knee arthrometer, d) the objective part of 2000 IKDC [30] knee examination form (surgical part) ranged from C level to D (indicating abnormal or severely abnormal), e) no participation in systematic recreational or sports activities and their activity level was assessed by Tegner activity score questionnaire [4] and ranged from 0–5 level and f) they were in the sub-acute phase of ACL injury – forty days to six months following ACL rupture [31,32].

Subjects' characteristics and inclusion criteria are shown in Table 1.

**Table 1:** Subjects' physical characteristics and admission criteria

**Subjects characteristics**	**E1–3 d/w, (n = 14)**	**E2–5 d/w, (n = 14)**	**C, (n = 14)**
	**Mean ± SD**	**Mean ± SD**	**Mean ± SD**
**Age (yrs)**	23.64 ± 2.56	25.07 ± 2.40	23.14 ± 2.71
**Weight (kgr)**	81.28 ± 8.40	82.50 ± 9.83	75.00 ± 8.00
**Height (cm)**	179.07 ± 5.18	182.21 ± 4.70	175.85 ± 5.78
**BMI (kgr/m^2 ^)**	24.80 ± 2.20	25.24 ± 2.90	25.80 ± 4.73
**Time of ACL injury (months)**	4.42 ± 1.79	4.42 ± 1.75	3.67 ± 1.78
SD (KT-1000)* (mm)	5.57 ± 2.40	6.35 ± 1.21	5.92 ± 2.12
**Tegner activity level (0–10)**	3.07 ± 1.32	3.28 ± 1.32	2.92 ± 1.43

Abbreviations: E1 (3 days/week) first experimental group (n = 14); E2 (5 d/w) second experimental group (n = 14); C control group (n = 14).

*Side-to-side difference: (SD) of tibial anterior translation on the injured side in mm.

BMI: body mass index.

Fifty eight subjects were initially assessed and excluded if they had a positive varus/valgus laxity test or they had a known meniscus injury that needed surgery. According to doctor's decision sixteen subjects were excluded because some of them needed meniscus surgery combined with the ACL reconstruction and the rest suggested to follow conservative rehabilitation program due to their positive varus knees. Other exclusion criteria included painful knee active range of motion, joint swelling, leg length discrepancy, and a history of lower extremity pain in the last six months that was not related to ACL.

This study was conducted in the General Army Hospital "401" (GAH 401).

The study received ethical approval from the Laboratory for Research of Musculoleskeletal system at the University of Athens. All subjects signed informed consent forms before participating.

### Operative technique

An arthroscopically assisted autograft technique was used in all subjects, using the semitendinosus and gracilis tendons (hamstring tendons- HT) as a graft source [33]. The placement of the graft was done by interference screw fixation of a four-stand hamstring graft [33].

The same surgeon performed all ACL reconstructions for this study.

### ACL traditional rehabilitation program

All subjects followed the traditional rehabilitation program for ACL reconstruction that was based on Wilk, et al. [34] and Majima, et al. [35] rehabilitation principles for hamstrings and gracilis graft (Table 2).

**Table 2:** ACL post-operative rehabilitation program based on hamstring tendon autograft (Wilk, et al. 2003; Majima et al. 2002).

**Post operative phase**	**Rehabilitation regimen**
Phase 1. Duration 2–4 weeks	Immediate straight leg raising. Early range of motion exercise with an emphasis on gaining full knee extension (0°). Weight bearing full as tolerated. First week 70° of flexion. Static squat (90° flexion)
Phase 2. Duration 2–3 months	Endurance training (biking). Progressive resistance training (leg press, calf press, step up). Dynamic squat (0°–110°). Balance exercises. Eccentric muscle contractions. Progressive resistance exercise full range of motion, hop on one leg without pain. Isokinetic exercise and assessment.
Phase 3. Duration 3–6 months	Continued progressive resistance and endurance training. Jogging/running, swimming. Eccentric training (active lengthening force production- such as jumping exercises). Strengthening and functional exercise training to prepare the individual for full return activity. Criteria for returning to full activity: 80% strength and 85% functional ability, proprioception > 90%, extension/flexion strength difference > 70% compared to the non-surgical lower extremity ysholm knee score > 90
Functional brace	6 weeks

ACL rehabilitation program, in this study, was the same for all subjects.

All subjects commenced the rehabilitation program one week following reconstruction and received the traditional ACL program five days per week (from Monday to Friday) for eight weeks. The program was delivered by two experienced physical therapists of the physiotherapy department of 401 GAH specializing in musculoskeletal conditions (mean experience in musculoskeletal physical therapy at least five years).

Prior to the commencement of the study the principal investigator was trained for a day separately to the physical therapist involved, in order to review and standardize the rehabilitation protocol procedure. The rehabilitation procedure between the physical therapists was blinded. All patients were instructed by their physical therapists to wear their functional brace and use crutches for six weeks during their daily activities. In order to ensure that all patients received similar amounts of exercise, a home exercise program was not given, and exercise level was monitored by the physical therapists verbally via standard questions which they asked all patients prior to every treatment session. Questions involved information about their current state (i.e. joint effusion, any pain etc.), as well as activities they performed between the treatment session, thus, enabling some monitoring of the patients' activities. Indeed, all patients complied with this program's routine.

However, the criteria of isokinetic assessment (following 8 weeks of rehabilitation) was identical for all patients, and comprised the following: no pain (indicated by a 0 on a 10 cm visual analog scale), no effusion (as measured by joint circumference), walking independently, 0° to 100–120° knee motion, straight leg raising in all planes, low resistance (10 reps) and multiple reps [20] with no extension lag and mini-squats 0°–100°.

### Cross eccentric exercise (CEE)

Cross training was an eccentric exercise program applied on the quadriceps' uninjured knee and based on previous studies [20,22,28]. Cross eccentric exercise started concurrently with the ACL physiotherapy program and was monitored by the same physical therapist.

Quadriceps strength of the uninjured knee was determined by one repetition maximum (1RM) in eccentric contraction on the isotonic (concentric/eccentric) leg extension machine. Subject was positioned on the leg extension device and the anatomical axis of the knee was aligned with the mechanical axis of the device [20,22]. Resistance was provided by a lever arm which was placed just above the medial malleolus.

Eccentric exercise program consisted of two to three warm up sets with no loads and followed by five sets of six repetitions (knee extension to flexion) at 80% intensity of 1RM of eccentric quadriceps strength [20,22,36] and two minutes' rest was allowed between each set. It has been reported [24-27,36] that quadriceps training with submaximal eccentric actions causes greater and faster strength adaptations than training with maximal ones does.

Thereby, intensity was kept constant throughout the eight weeks period in order to simplify, facilitate standardization and clinical applicability of the procedure. The CEE program was not differentiated throughout the training period for any of the experimental groups in order to monitor all patients. So, the resistance utilized ranged from 60.85 ± 13.93 kg for the first and 61.50 ± 11.40 kg for the second experimental group. Subjects performed each eccentric contraction with one leg-the uninjured one.

Both experimental groups performed 8 weeks of CEE. The first 8 weeks following the implantation is critical as the hamstring tendon graft increases in strength and stiffness [7,33-35], and the muscle follows specific biochemical, mitochondrial and neurological adaptations [36,37]. However, the eight weeks duration has been considered as a critical time in establishing a strength stimulus on weak quadriceps muscle following ACL reconstruction.

The first experimental group followed the CEE at a frequency of three days per week (E-3 d/w), and the second experimental group followed the CEE at five days per week (E-5 d/w). Patients from the two experimental groups participated at their CEE when the ACL rehabilitation program was completed.

## Main outcome measures

Evaluation procedure was identical for all subjects, and was carried out by the same examiner. The evaluation procedure was blinded and conducted by a Kin Com AT^+ ^isokinetic machine. The reliability of the Kin Com AT^+ ^isokinetic dynamometer as an evaluating tool for measuring muscle strength parameters has been well established in previous studies [38,39].

A pilot study was conducted before testing and based on the number of repetitions necessary to produce reliable scores.

All subjects were evaluated on quadriceps accelerated reaction time (ART) or time to peak in two phases: three days pre-operatively (pretest) and eight weeks post-operatively (posttest). Quadriceps ART was evaluated by isometric contraction at the angles 45°, 60° and 90° of knee flexion at both knees [39].

ART outcome measure was considered as impairment on quadriceps muscle performance after the ACL reconstruction. Subjects were positioned in a seated position, with the hips and knees at 90° flexion and the thighs, pelvis and upper body firmly strapped to the seat of the dynamometer.

Prior to testing, a warm up consisting of five minute stationary bicycle at self selected sub-maximal intensity was completed.

Knee static angles were set by the dynamometer. Each subject performed three maximum isometric contractions of 5 seconds duration for both phases (pretest and posttest). Subjects were given visual and verbal encouragement. The uninjured knee was tested first followed by the ACL injured one. Peak ART value of each repetition and each angle was averaged and used for statistical analysis.

The Lysholm questionnaire was included as a disability outcome measure following ACL injury and reconstruction. The rating system of Lysholm questionnaire has been well established, as an alternative mechanism to gather outcomes data when evaluating knee ligament injuries [4,40]. The questionnaire has a total score of 100 points and consists of the following variables: Limping, crutch support, knee instability, knee locking, pain, swelling, knee function with stair climbing and knee function with squatting [4].

All subjects in the three groups completed the questionnaire in two phases: three days pre-operatively (pretest) and eight weeks post-operatively (posttest). The total score of each subject pre and post-operatively was used for statistical analysis.

## Statistical analysis

### Data were analyzed with SPSS software

To account for pretest differences of quadriceps ART scores among the groups on the ACL injured knee, analysis of Covariance (ANCOVA) was applied to the dependent variable-quadriceps ART posttest scores at 45°, 60° and 90° of knee flexion.

Two factor ANOVA (group × time) was applied to test group differences for the dependent variables quadriceps ART at 45°, 60°and 90° respectively, where the group factor had three levels (C, E1–3 d/w, E2–5 d/w), and the time factor had two levels (pre-operatively and post-operatively). Two factor ANOVA (group × time) was used to assess group differences in Lysholm scores pre-operatively and post-operatively. Post hoc analysis based on Tukey HSD criterion was applied to determine the location of group differences after a significant F, on the ACL injured knee.

Level of statistical significance was set at 0.05.

## Results

There were no differences among the groups in baseline physical characteristics.

Mean and standard deviation of ART at 45°, 60° and 90° of flexion, and the subjective Lysholm scores (SLS) on ACL injured knee, for all groups are shown in Tables 3 and 4 respectively.

**Table 3:** Mean and standard deviation (Mean ± SD) values of quadriceps accelerated reaction time (ART) (sec) for the two phases of evaluation on ACL injured knees.

	**Pretest evaluation**			**Posttest evaluation**			
**Angles**	**E1(3 d/w)**	**E2(5 d/w)**	**C**	**E1(3 d/w)**	**E2(5 d/w)**	**C**	**Overall significance (Two-way ANOVA)**
45°	2.72 ± 1.01	2.83 ± 1.12	2.35 ± 1.23	3.05 ± 0.90	2.90 ± 1.13	2.88 ± 0.68	0.62 NS
60°	2.79 ± 0.73	2.75 ± 1.13	2.82 ± 1.16	2.82 ± 0.70	3.45 ± 0.96	3.27 ± 0.99	0.74 NS
90°	2.50 ± 1.07	2.67 ± 1.07	3.21 ± 1.05	2.45 ± 0.73	3.37 ± 1.02	3.42 ± 0.82	0.02* S

Abbreviations: E1 (3 days/week) first experimental group (n = 14); E2 (5 d/w) second experimental group (n = 14); C control group (n = 14).

Statistical level *P *< 0.05, S = significance*, NS = no significance

**Table 4:** Mean and standard deviation (Mean ± SD) of patients' Lysholm knee scores (SLS) between the two phases of evaluation on ACL injured knee, for all three groups.

**Groups**	**Pretest evaluation**	**Posttest evaluation**	**Overall significance (Two-way ANOVA)**
**E1(3 d/w)**	83.92 ± 6.86	92.28 ± 4.35	
**E2(5 d/w)**	78.00 ± 9.70	90.57 ± 6.16	0.01*S
**C**	76.00 ± 9.70	84.78 ± 6.91	

Abbreviations: E1 (3 days/week) first experimental group (n = 14); E2 (5 d/w) second experimental group (n = 14); C control group (n = 14).

Statistical level *P *< 0.05, S = significance*

ANCOVA showed no statistical significant effect of the covariate-pre-test ART scores at 45° and 60° on the dependent variable ART post-test scores. ANCOVA revealed a statistical significant effect of the covariate-pre-test ART scores at 90° (F = 4.64, p < 0.01) on the dependent variable ART post-test scores.

This means that the ART post-test means of the groups were influenced by their pre-test ART scores (R^2 ^= 0.21).

Two factor ANOVA did not show statistical significant differences among the groups for the variable ART at 45° (F = 0.39, p = 0.67, p > 0.05) and 60° (F = 0.10, p = 0.75, p > 0.05) of knee flexion. On the other hand, statistical significant differences were shown for ART at 90° among the groups (F = 4.29, p = 0.02, p < 0.05) (Table 3).

**Figure 1:**
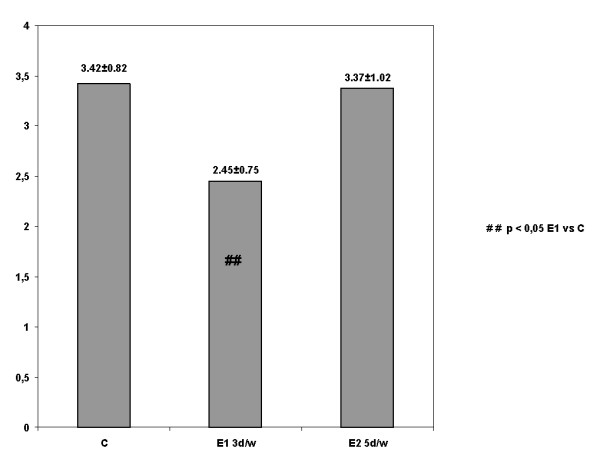
**The effect of CEE on quadriceps ART in ACL reconstructed knee at 90° that determines the significant differences between the first experimental group in comparison with the control (*Tukey HSD, Post hoc analysis*)**.

Post hoc analysis by Tukey HSD determined that the above significant results arose from the first experimental group in comparison with the control (D = -0.83, p = 0.01) (Figure 1). No significant differences on ART were observed between the two experimental groups and, between the second experimental and the control group following eight weeks of CEE (Table 3).

ANCOVA revealed a statistical significant effect of the covariate-pre-test SLS (F = 9.10, p < 0.01) on the dependent variable post-test SLS. This shows that the post-test SLS means of the groups were influenced by their pre-test SLS (R^2 ^= 0.37).

**Figure 2:**
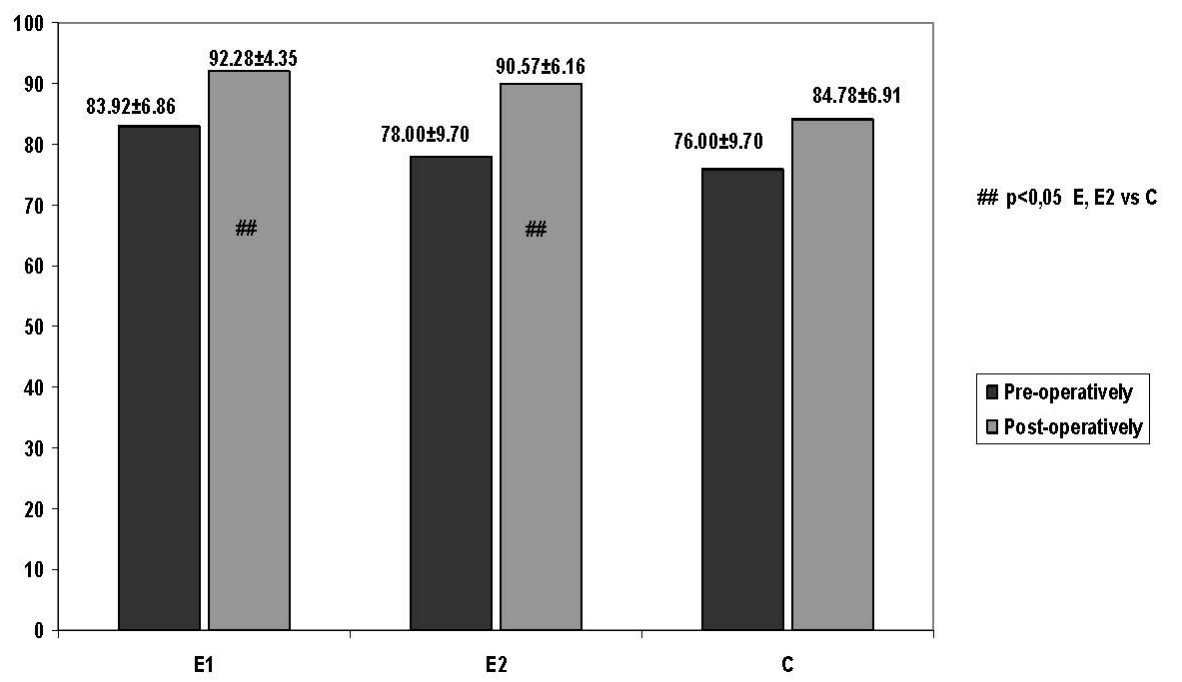
**The effect of CEE on subjective scores (SLS) in ACL reconstructed patients that determines the significant differences between the first and second experimental groups in comparison with the control (*Tukey HSD, Post hoc analysis*)**.

Two factor ANOVA revealed statistical significant differences for SLS among the groups (F = 4.75, p = 0.01, p < 0.05) (Table 4). Post hoc analysis by Tukey HSD determined that the above significant results arose from the first experimental group in comparison with the control (D = 7.5, p < 0.01) and from the second experimental group in comparison with the control (D = 3.78, p = 0.03) (Figure 2). No significant differences on SLS were observed between the two experimental groups (Table 4).

## Discussion

The results of this study supported our hypotheses that adding cross eccentric exercise to the traditional ACL rehabilitation program would be more beneficial on quadriceps accelerated reaction time and less disabling on the ACL reconstructed patients.

According to the primary objective of this study which investigated the effect of cross eccentric exercise (CEE), applied three and five days per week, on the quadriceps accelerated reaction time (ART) at the angles 45°, 60° and 90° of knee flexion, the results were statistically significant only at 90° in the early rehabilitation phase of ACL reconstructed knee.

This is possibly attributed to the relationship between ACL and anterior tibial translation and quadriceps muscle activity at 90° of knee flexion that causes less displacement of the tibial [5-14]. To our knowledge, previous literature has not investigated the effect of CEE on quadriceps ART in ACL reconstructed patients. Therefore, these results seem to support the concept that CEE could be included as an essential element to improve the ART of quadriceps after the ACL reconstruction. ART of quadriceps muscle is an important factor against knee joint injuries because joint loading especially in sports activities, requires fast and coordinated muscle action. Further research needs to be conducted giving the potential to work dynamically in muscles – following eight weeks of ACL reconstruction- to generate rotational torque of the tibial.

CEE produced more improvement on quadriceps ART in the first experimental group (E1–3 d/w) in comparison with the control group. On the other hand, no significant differences were found between the experimental groups and between the second experimental (E 5 d/w) and the control group.

In trying to investigate the most effective CEE frequency for improvement of the quadriceps ART on the ACL reconstructed knee, two sets of frequencies were explored; 3 and 5 days per week for the first and second experimental group respectively. The decision on the above frequencies was based on the fact that any exercise training program must be performed for a sufficient frequency and duration in order to allow the muscle specific biochemical, mitochondrial and neurological adaptations to take place [36,37,41].

Although, a positive effect of CEE was found following ACL reconstruction, it is unclear how the CEE mode of training was responsible for the results observed in this study. For example, the group receiving less training (3 d/w) did better. These statistical results may be attributed to the fact that the rest between the days of training was important for the appropriate neuromuscular adaptations to occur. The benefits of prolonged training sessions in enhancing performance may be more related to adaptations in cardiovascular functions (which are not directly related to muscle specific adaptations) [36,41].

As CEE in different frequencies has never been explored in ACL patients before, definite conclusions cannot be made and thereby, no reports in the literature have showed whether any particular frequencies can affect quadriceps performance [17]. Further research should explore different exercise frequencies in cross exercise for this patient population.

According to the second purpose of this study which investigated the effect of CEE, applied three and five days per week, on a disability questionnaire, significant results were shown on subjective scores of disability in ACL reconstructed patients.

Previous literature has considered subjective scores appropriate as pre-operative and post-operative indicators of disability incorporated with other objective factors for ACL patient's evaluation [1-5]. On the other hand, the effect of cross exercise has never been investigated as one of the factors that could influence knee disability following ACL injury. Therefore, the above finding presents a new research field that of cross exercise effect and its relationship to performance of daily activities and muscular characteristics in ACL rehabilitation progression.

As far as investigating the most effective frequency of CEE, significant improvements appeared in both experimental groups in comparison with the control group. No significant differences were found between the experimental groups.

These statistical results may be attributed to the fact that patients who followed a supplementary rehabilitation program of CEE at a sequence of 3 and 5 days per week felt that the amount of post-operative rehabilitation could be more efficient. From a rehabilitation perspective it would appear logical that a reinforced rehabilitation program could give better results.

In terms of the clinical applicability, these findings provide valuable and possibly promising information about the effect of cross eccentric exercise in the early phase of ACL reconstruction. Irrespective of the statistical significant results of this study the experimental groups-following cross exercise-showed more improvement than the control group that did not follow CE and this conclusion is of clinical significance.

## Limitations

A few limitations characterize the current study. We did not determine the effectiveness of CEE following ACL reconstruction for the dominant and non-dominant limb due to our small sample size. Future studies are needed to clarify this issue.

An additional limitation was that, the three groups did not seem to have the same performance level at the beginning of this study. Therefore, we analyzed the effect of the pre-test quadriceps scores on the post-test scores at both variables -ART and Lysholm knee scores.

The results revealed that the post-test means were influenced by the pre-test scores. However, no statistically significant differences were found among the groups on the pre-test quadriceps scores.

## Conclusion

These preliminary findings provide some evidence that adding CEE as an adjunct to a traditional rehabilitation program improves quadriceps accelerated reaction time at 90° of knee flexion on the ACL reconstructed knee.

CEE at the frequency of 3 and 5 d/w induced better subjective scores, according to daily activities performance on ACL reconstructed patient's compared to the control group.

Finally, the control group (which followed only the traditional rehabilitation for ACL reconstructed knee), had less improvement in comparison with the experimental groups in terms of quadriceps accelerated reaction time and subjective scores on ACL reconstructed patient's (Lysholm questionnaire), thus, supporting the remarkable role of cross exercise.

## Competing interests

The authors declare that they have no competing interests.

## Authors' contributions

MP: served as a project lead, contributing substantially to conception, design, and collection of data, analysis and interpretation of data, and wrote the fist draft of the paper, EB: has been involved in collecting the data and in substantially revising the manuscript, EA: has been involved in the acquisition of the data, NP: has provided the general supervision of its design. All authors read and approved the final manuscript.
